# Sorafenib-induced Acute on Chronic Liver Failure in a Patient with Hepatocellular Carcinoma After Microwave Ablation

**DOI:** 10.7759/cureus.5176

**Published:** 2019-07-19

**Authors:** Anam Naveed Malik, Asim Tameez Ud Din, Farooq Mohyud Din Chaudhary, FNU Quratulain, Khaleeq H Siddiqui

**Affiliations:** 1 Internal Medicine / Gastroenterology, Nishtar Medical University & Hospital, Multan, PAK; 2 Internal Medicine, Rawalpindi Medical University, Rawalpindi, PAK; 3 Gastroenterology, Nishtar Medical University & Hospital, Multan, PAK; 4 Internal Medicine, NewYork-Presbyterian Queens, Flushing, USA

**Keywords:** sorafenib, acute on chronic liver failure, microwave ablation

## Abstract

Hepatocellular carcinoma (HCC) is becoming a rapidly prevalent hepatic tumor throughout the world. Initially, liver transplantation and resection were the only available options. But there is a recent advent of new treatment modalities like ablative embolization techniques and chemotherapy. Guidelines are available regarding the use of these techniques according to the stage of the tumor. Sorafenib is a chemotherapeutic agent approved for the management of advanced HCC. It works by inhibiting different tyrosine kinases, which halt the progression of the tumor. The common side effects associated with it are diarrhea, hand-foot skin reaction, and alopecia. Acute on chronic liver failure (ACLF), defined as the development of acute liver failure, in the setting of chronic liver disease, is a rare adverse event associated with sorafenib. Here, we present a case of a 65-year-old male presented to Nishtar Hospital Multan, Pakistan, who developed advanced-stage HCC due to underlying liver cirrhosis. There was no metastasis or vascular involvement. After discussing the options, he selected microwave ablation (MWA). There was a recurrence of the tumor after the procedure so he was started on sorafenib. A week after the initiation of a low dose drug (200 mg twice daily), he developed signs and symptoms of ACLF, which included hyperbilirubinemia, prolonged prothrombin time (PT), and flapping tremors. He was admitted to the intensive care unit (ICU) and was successfully managed. He was discharged with a follow-up scheduled after two weeks. This is a unique and rare adverse event of sorafenib.

## Introduction

The prevalence of hepatocellular carcinoma (HCC) is rising all around the world. A study reported about 14 million cases in 2012, which is expected to rise in the coming years, making it one of the most common primary hepatic tumors [1]. Multiple treatment options are available, depending upon the stage of the tumor. Liver transplantation, although one of the curative options, is not feasible in many patients because of the late presentation of HCC. Currently, ablative techniques, including radiofrequency ablation (RFA) and microwave ablation (MWA), are also being used. Other options include surgical resection, embolization techniques, and systemic chemotherapy. Sorafenib is an approved chemotherapeutic agent for HCC. It works by inhibiting certain tyrosine kinases that are involved in HCC tumor progression and vascular growth [2-3]. A phase III trial done to assess the efficacy and safety of sorafenib demonstrated liver dysfunction in <1% of the patients in the treatment group [4].

Acute on chronic failure (ACLF), as implied by its name, is defined as the acute development of liver dysfunction in the setting of chronic liver disease [5]. Here, we report the case of an HCC patient who was diagnosed with ACLF after initiating sorafenib following MWA.

## Case presentation

A 65-year-old male presented to Nishtar Hospital Multan, Pakistan, in 2016, with the complaint of multiple episodes of hematemesis. There was no history of viral hepatitis, alcohol intake, diabetes, or any other co-morbid illness. There was no significant family history of similar illness or liver disease. Examination showed pallor, vitiligo, and palmar erythema. Flapping tremors were absent. Abdominal examination showed an enlarged spleen. Further workup revealed hemoglobin 7.3 g/dl (normal 13-18 g/dl), platelet count 120,000/mm^3^ (normal 150,000-400,000 /mm^3^), albumin 3.1 g/dl (normal, 3.5-5.5 g/dl), total bilirubin 1.5 mg/dl (normal up to 1.2 mg/dl), aspartate aminotransferase (AST) 51 U/l (normal range, 10-40 U/l), and alanine aminotransferase (ALT) 68 U/l (normal range, 7-56 U/l). Prothrombin time was 15 sec (control 12 sec). Hepatitis B surface antigen (HBsAg) and antibodies to hepatitis C virus (anti-HCV) were negative. Ultrasound showed coarse echotexture of the liver with irregular margins. However, no lesion was seen. Mild ascites was noted, and the spleen was enlarged. A Child-Pugh score of 7 (Class B) was calculated based on the above parameters. The patient was resuscitated with vasopressors (octreotide) and packed red blood cells (RBCs). After hemodynamic stability, esophagogastroduodenoscopy (EGD) was performed, which showed esophageal varices with stigmata of a recent bleed. Band ligation was performed. A final diagnosis of decompensated chronic liver disease complicated by variceal bleed was established, and the patient was discharged upon beta-blockers, spironolactone, and lactulose.

Afterward, the patient did not get proper follow-up for an evaluation of the cause of his liver disease. He later developed an episode of portosystemic encephalopathy (PSE) and worsening of ascites in 2017. He was on beta-blockers and diuretics, with poor compliance.

**Figure 1:**
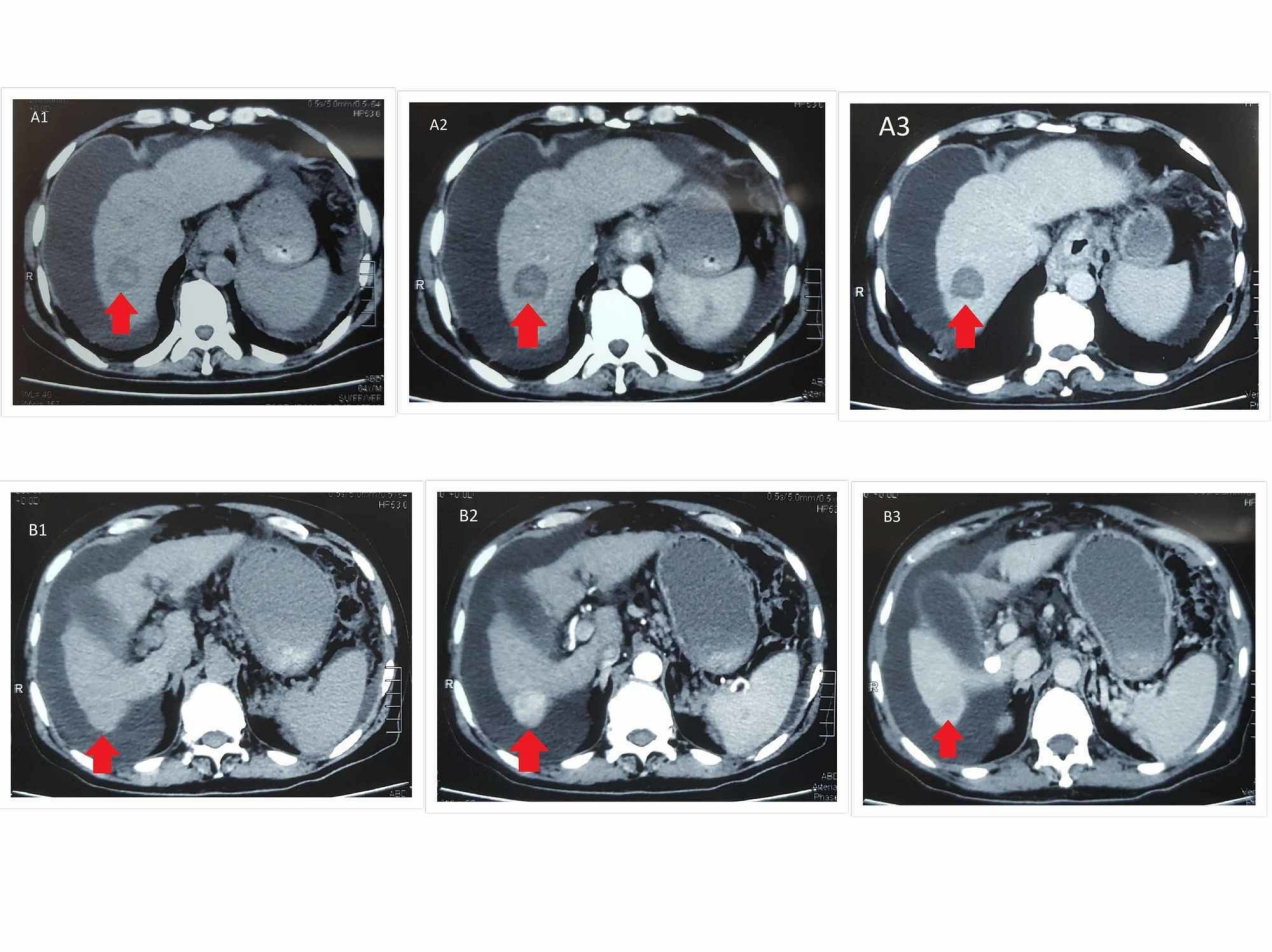
Triphasic CT abdomen Showing one lesion in segment VIII (A) and another in segment VI (B) of the liver in successive plain (A1/B1), arterial (A2/B2), and venous (A3/B3) phases of the study. A hypodense lesion (A1) in segment VIII was consistent with previous successful microwave ablation. This lesion is neither showing arterial hyperenhancement (A2) nor any washout (A3). A new arterialized lesion (B2) is seen in segment VI of the liver, showing washout of contrast on the venous phase (B3). No evidence of thrombosis in the portal vein or extrahepatic metastasis seen. Gross ascites is also evident. CT: computed tomography

In 2019, he complained of fatigue and started losing weight, which prompted him to consult a physician in a private hospital of Multan. An ultrasound abdomen in March 2019 revealed a hypoechoic mass of about 3.5 x 2.6 cm. Subsequently, triphasic computed tomography (CT) abdomen revealed a single 3.5 x 4.2 cm lesion in segment VIII of his liver, which showed arterial hyper-enhancement with washout in the venous phase. Mild ascites was noted. There was no metastasis, and the portal and hepatic veins were patent. Labs revealed Hb 11.5 g/dl, albumin 2.9 g/dl, bilirubin 2.8 mg/dl, PT 18 sec (control 13 sec), HBsAg negative, and anti-HCV negative. At that time, his Child-Pugh score was B9. His Barcelona Clinic Liver Cancer (BCLC) stage was advanced (stage C) due to deranged liver function. He was advised a live donor liver transplant (LDLT). However, he was not willing for LDLT and inquired about alternative management options. He underwent microwave ablation (MWA) in April 2019. A post-procedure scan showed successful ablation of the lesion. He tolerated the procedure well and was discharged. Follow-up was planned after four weeks. On follow-up, triphasic CT abdomen in May 2019 showed a hypodense lesion 2.5 x 2.5 cm in segment VIII, consistent with previous successful MWA as well as a new arterialized lesion in segment VI of size 2.7 x 2.3 cm showing washout of contrast on venous phases (see Figure 1). No evidence of thrombosis in the portal vein or extrahepatic metastasis was seen. Gross ascites was also evident at that time.

Following the diagnosis of advanced recurrent HCC, the patient was then started on tab sorafenib 200 mg twice daily in June 2019. A week after starting the new drug, the patient developed nausea, vomiting, loose motions, and yellow discoloration of the eyes. He was admitted to the intensive care unit of Nishtar Hospital Multan. Upon admission, the patient had fever 100°F, jaundice, clubbing, vitiligo, and palmar erythema. He was slightly confused. Flapping tremors were present. Abdominal examination showed a mildly tender distended abdomen with reduced liver span and ascites. Labs were as follows: hemoglobin 11.9 g/dl with a hematocrit of 32.5 and mean corpuscular volume (MCV) of 100 fL, total leukocyte count (TLC) 8000/mm^3^, platelets 71000/mm^3^. He had hypoalbuminemia (3.2 g/dl, normal 3.5-5), PT 30 sec (control 12 sec), and international normalized ratio (INR) 2.5. Total bilirubin on the day of admission was 3.5 mg/dl, which rapidly increased to 17.8 mg/dl after two days, with ALT 17.4 and AST 79.4, urea 29 mg/dl, creatinine 0.55 mg/dl, sodium 132 mEq/l, potassium 4.03 mEq/l, alpha-fetoprotein 132 ng/ml (normal 0-10 ng/ml). His Child-Pugh score was 13, model end-stage liver disease (MELD) 17, and MELD sodium (MELD-Na) 31. Ascitic fluid examination showed a high serum ascites albumin gradient (SAAG) ascites without evidence of spontaneous bacterial peritonitis (SBP). HBsAg and Anti-HCV were negative. Antibodies to hepatitis A and E of the immunoglobulin M (IgM) variety were negative. Hepatitis B core IgM was also negative. There was no history of illicit or indigenous drug usage as well as any alcohol intake.

These results were consistent with the diagnoses of ACLF. After ruling out the typical causes, the etiology of acute insult was determined to be the newly started drug sorafenib. The patient was managed in the intensive care unit with intravenous dextrose, containing fluids, antibiotics, and lactulose. After a few days, his condition started improving. His jaundice and coagulopathy started improving gradually. Eventually, he was discharged with a plan to follow-up in two weeks' time to assess recovery and discuss future treatment options.

## Discussion

Multiple modalities are available for the management of HCC, including liver transplant, resection, embolization, ablation, chemotherapy, and supportive care. The treatment mainly depends on the grade and stage of the tumor. Liver transplantation is a curative treatment for early-stage liver tumors [6]. Although our patient had advanced liver disease (stage C), he was advised LDLT, as there was no metastasis or vascular invasion visible on triphasic CT scan. Our patient agreed to MWA but there was a recurrence of the tumor.

Sorafenib is approved for the treatment of advanced HCC [7]. Based on these findings, our patient was started on sorafenib 200 mg twice daily. Although the recommended dose is 800 mg/day (400 mg twice daily), studies have found that there is no difference in the two regimens in terms of efficacy. It is recommended to start with a low dose in patients who are prone to develop complications, which was the case with our patient [8].

The most common adverse effects associated with sorafenib are loose stools, hand-foot skin reaction, and alopecia. Our patient also showed some of these symptoms. Liver failure was not reported as a major adverse event in clinical trials [4,7]. Wang QL et al. reported a case of ACLF induced by sorafenib after trans-arterial chemoembolization (TACE) and RFA, and the dose of the drug was 400 mg twice daily [9]. Van Hootegem A et al. also reported a similar case [10]. In contrast to these two cases, our patient underwent MWA, and the dosing schedule was 200 mg twice daily.

ACLF is defined in multiple ways in different studies. The common points include the manifestation of acute signs of liver failure in a patient with a chronic liver disease, most often preceded by a precipitating event. The acute signs of failure mentioned in the literature are raised bilirubin, deranged liver function tests, and prothrombin time (PT) [11-13]. In this case, the total bilirubin was 3.5 mg/dl on admission, which raised to 17.8 mg/dl in two days and PT was 30 seconds.

The management of ACLF is mainly supportive. Other specific symptoms like hepatic encephalopathy, raised intracranial pressure, and coagulopathy should be treated according to guidelines. In the present case, the patient was managed similarly and was discharged after an improvement in symptoms [14].

## Conclusions

ACLF is a rare but serious adverse event associated with sorafenib in a patient with advanced HCC. It is diagnosed primarily by the clinical presentation and laboratory values. Jaundice and prolonged PT are common manifestations. The management is mainly supportive. In this report, we present a case of HCC, which was unsuccessfully ablated by microwave ablation. Sorafenib was started due to its advanced stage but signs of acute liver failure were developed within a week of initiation of therapy. This is a very rare adverse effect of sorafenib.
